# Oxidative stress and hepatitis C virus

**DOI:** 10.1186/1743-422X-10-251

**Published:** 2013-08-07

**Authors:** Usman Zafar Paracha, Kaneez Fatima, Mohammad Alqahtani, Adeel Chaudhary, Adel Abuzenadah, Ghazi Damanhouri, Ishtiaq Qadri

**Affiliations:** 1 Department of Pharmaceutics, Hajvery University, Lahore, Pakistan; 2 IQ Institute of Infection and Immunity, Lahore, Punjab, Pakistan; 3 Center of Excellence in Genomic Medicine, King Abdul Aziz University, PO Box 80216, Jeddah, 21589, Saudi Arabia; 4 Faculty of Applied Medical Sciences, King Abdulaziz University, PO Box 80216, Jeddah 21589, Saudi Arabia; 5 King Fahd Medical Research Center, King Abdul Aziz University, PO Box 80216, Jeddah 21589, Saudi Arabia

**Keywords:** Oxidative stress, ROS, HCV

## Abstract

The disproportionate imbalance between the systemic manifestation of reactive oxygen species and body’s ability to detoxify the reactive intermediates is referred to as oxidative stress. Several biological processes as well as infectious agents, physiological or environmental stress, and perturbed antioxidant response can promote oxidative stress. Oxidative stress usually happens when cells are exposed to more electrically charged reactive oxygen species (ROS) such as H2O2 or O2-. The cells’ ability to handle such pro-oxidant species is impeded by viral infections particularly within liver that plays an important role in metabolism and detoxification of harmful substances. During liver diseases (such as hepatocellular or cholestatic problems), the produced ROS are involved in transcriptional activation of a large number of cytokines and growth factors, and continued production of ROS and Reactive Nitrogen Species (RNS) feed into the vicious cycle. Many human viruses like HCV are evolved to manipulate this delicate pro- and antioxidant balance; thus generating the sustainable oxidative stress that not only causes hepatic damage but also stimulates the processes to reduce treatment of damage. In this review article, the oxidant and antioxidant pathways that are perturbed by HCV genes are discussed. In the first line of risk, the pathways of lipid metabolism present a clear danger in accumulation of viral induced ROS. Viral infection leads to decrease in cellular concentrations of glutathione (GSH) resulting in oxidation of important components of cells such as proteins, DNA and lipids as well as double strand breakage of DNA. These disorders have the tendency to lead the cells toward cirrhosis and hepatocellular carcinoma in adults due to constant insult. We have highlighted the importance of such pathways and revealed differences in the extent of oxidative stress caused by HCV infection.

## Background

In biological system, the oxidative stress refers to the physiological disturbance between the ROS such as H2O2 or O2- and the ability of the body to remove them. Oxidative stress also promotes nitrosative stress caused by reactive nitrogen species resulting in perturbed cellular signaling and cellular damage. Some of the viral infections such as HCV infections decrease the cell’s ability to work against such pro-oxidant species especially in liver [1]. Oxidative stress can also be defined as the disordered redox signaling and control [2].

A variety of ROS are produced throughout the body, which are found to be the by-products of cellular metabolism, and play an important role in cell signaling and regulation of cytokine, growth factor and hormone action, transcription, ion transport, neuromodulation, immunemodulation and apoptosis [3,4]. In particular, they play a fundamental role in normal functioning of immune system and proliferation of T cells and immunological defence [5,6].

One particular species of interest; superoxide (O2-), is generated either by accidental result of incomplete electron transfers in the electron transport chain or by design in activated white blood cells with the function of destroying pathogens. Upon production, O2- molecules are rapidly metabolized into hydrogen peroxide (H2O2), which further helps in destroying some pathogens. Intermediate concentrations of H2O2 (and certain other ROS) result in activation of nuclear factor κB (NF-κB), and activating protein-1 (AP-1), which are transcription factors that up-regulate several antioxidant pathways. They are neutralized by the activity of key antioxidant genes such as Manganese, Magnesium or Copper Superoxide dismutase (Mn, Mg or Cu-SOD) [7].

### ROS and oxidative stress

ROS are usually produced during the processes of aerobic metabolism, ongoing stress, and exposure to UV light or X-rays. They play an important role in many of the signaling reactions in different organisms from bacteria to mammalian cells. These were previously considered as only the toxic by-products but now they are also known to work in complex signaling network of cells [8].

Many different enzymes in mitochondria, endoplasmic reticulum, peroxisomes and other cell compartments are involved in synthesis of ROS [9,10] that causes cellular stress either through direct interaction with the biological molecules such as proteins, lipids and nucleic acids, or through activation of classical signaling cascades involved in stress responses including protein kinases, cytokines, and transcription factors [8,11] leading to inflammatory responses.

### Role of mitochondria and endoplasmic reticulum in oxidative stress

Mitochondria generate ROS as a by-product of ATP synthesis via oxidative phosphorylation that may cause oxidative injury to mitochondrial DNA (mtDNA) [12,13]. Different oxidative stress conditions usually result in various diseases associated with alteration or depletion of mtDNA copy numbers [14-17].

HCV also changes the steady-state levels of a mitochondrial protein chaperone, referred to as prohibitin, that disturbs the mitochondrial respiratory chain leading to overproduction of ROS [18].

Endoplasmic reticulum (ER) is an organelle in the cells that is responsible for folding of proteins. Researchers are of the opinion that protein oxidation in ER results in protein folding and production of ROS that results in oxidative stress. ER is also the primary storage site for calcium that is required for protein folding reactions [19]. Researchers reported that oxidative stress may result in elevated leakage of calcium from ER lumen and the same thing happens in ER stress [20-22]. As more calcium comes in cytosol, mitochondrial ROS production increases [19].

### Role of hepatitis C virus (HCV) in oxidative stress

Hepatitis C virus (HCV) belongs to Flaviviridae family of RNA viruses having positive strand RNA genome [23] of approximately 9400 bp in length [24]. HCV results in 3–4 million new cases of viral hepatitis annually. Nearly 150 million people are chronically infected having a risk of liver cirrhosis and/or liver cancer [25]. In the mid 1990s, researchers found the occurrence of oxidative stress during chronic hepatitis C [26].

Although viral replication mostly takes place in the hepatocytes but HCV potentially attacks the cells of the immune system and propagates there. In this case, lymphocytes are found to be involved in the occult – occult HCV infection (OHCI) – and active forms of disease. OHCI causes phosphoinositol 3-kinase-mediated cellular response in peripheral blood lymphocytes after mitochondrial oxidative stress and damage to DNA double strands. OHCI has been found to be related to increased risk of developing hepatocellular carcinoma and lymphoproliferative disorders [27].

Researchers reported that in chronic hepatitis, immunity initiates the production of ROS [28] and nitric oxide (NO) [29]. This was further reported by Farinati et al. that HCV produces more ROS than other hepatitis viruses [30] and patients with chronic hepatitis C have over 80% chances of developing chronic diseases (CHC) as compared to patients of hepatitis A, B and E [31]. An increase in the amount of ROS by two to five orders of magnitude in liver tissue from CHC patients have also been reported [32,33] and significant increase in lymphocytes of patients with chronic and OHCI [27].

HCV replicates in cytoplasm and results in chronic infections that may finally cause chronic hepatitis, cirrhosis, and hepatocellular carcinoma (HCC) [34,35]. The extent of mitochondrial injury and severity of oxidative injury exerted in liver tissue represent severity of HCV infection [36]. Oxidative stress has also been found to play an important role in HCV genome translation that is found to be mediated via PERK-mediated inhibition of cap-dependent translation [26]. Previously researchers found that ROS induced viral genome heterogeneity is the probable mechanism for viral escape from the immune system [37].

#### ***HCV proteins associated with oxidative stress***

HCV viral nucleocapsid protein, an HCV core protein, has been found to increase the oxidative stress in liver [38]. Out of 10 viral proteins, the core protein is the strongest regulator [39,40] but is not the only one associated with increased oxidative stress as NS3, or NS5A proteins have also been found to increase the oxidative stress [41-43]. In further studies, researchers found that E1 [39], E2 [29,44], and NS4B [39,45] are also involved in oxidative stress.

The nonstructural protein 5A (NS5A), which is an integral membrane protein important for replication of virus along with other important phenomenon such as interferon resistance, and apoptosis [46], encoded by human HCV RNA genome, changes the calcium levels. The core, NS5A, and NS3 proteins not only increase calcium uptake by mitochondria but also cause oxidation of mitochondrial glutathione leading to increased ROS [13,47,48] in mitochondria resulting in translocation of NF-κB and STAT-3 transcription factors into the nucleus leading to oxidative stress. Antioxidants remove NS5A-induced activation of NF-κB and STAT-3 [49,50]. NS4B also increases the translocation of NF-κB into the nucleus through protein-tyrosine kinase (PTK) mediated phosphorylation and subsequent degradation of κB alpha [45]. Basic points of viral proteins involved in oxidative stress are mentioned in the Table 1.

**Table 1:** HCV proteins involved in oxidative stress

**HCV proteins**	**What they can do?**
**Core protein, NS3, NS5A, E1, E2, NS4B**	• Increase oxidative stress
**Core protein, NS5A, NS3**	• Increase calcium uptake by mitochondria
	• Oxidation of mitochondrial glutathione leading to elevated ROS

NS5A protein has also been found to play an important role in activation of p38 MAPK (mitogen-activated protein kinase), JNK (c-Jun N-terminal kinase) and AP-1 (activator protein-1) that are linked to increased oxidative stress leading to increased MnSOD (manganese-superoxide dismutase) antioxidant responses [1].

**Figure 1:**
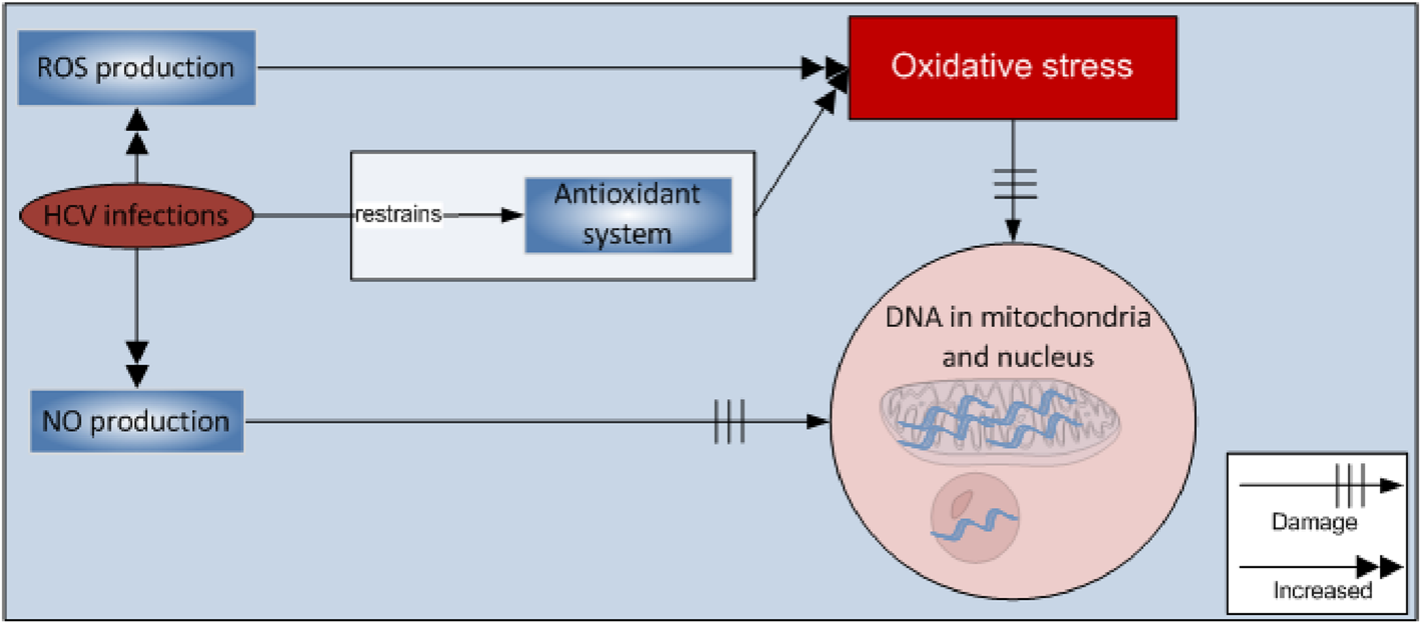
HCV infection and DNA damage.

Along with ROS, NO also causes oxidative DNA damage. NO not only damages DNA but also inhibits DNA repair [51-53] as shown in Figure 1. The Casein kinase 2 and phosphoinositide-3 kinase mediates the effect of core and NS5A proteins [39].

#### ***Effect of HCV on the enzymes***

Research showed increased levels of some of the defense enzymes such as heme oxygenase (HO-1) [54] and thioredoxin (Trx) [55,56] in patients of CHC. HCV also poses danger to antioxidant systems in the body such as HO-1 and NADH dehydrogenase quinone 1 [18] that may lead to increased oxidative stress in liver during infections caused by HCV. In addition to these, researchers found decreased levels of many other antioxidant defense enzymes, such as manganese or Cu/Zn superoxide dismutase (SOD), glutathione reductase, and glutathione peroxidase, in the peripheral blood mononuclear cells (PBMC) of the patients of CHC [27,57,58].

HCV induces the expression of 3β-hydroxysterol Δ24-reductase (DHCR24) as 5′-flanking genomic promoter region of DHCR24 is responsive to HCV. This region also binds Sp1 transcription factor in response to oxidative stress under the regulation of ataxia telangiectasia mutated (ATM) kinase. Overexpression of DHCR24 decreases acetylation resulting in disturbed p53 activity leading to suppressed hydrogen peroxide-induced apoptotic response in hepatocytes [59].

HCV also acts as a regulator of Nox4, a member of the NADPH oxidase (Nox) family, inducing ROS production through autocrine transforming growth factor β (TGF-β)-dependent mechanism [60]. ROS are found to have an important influence on development of inflammatory liver disease mediated by HCV [61,62].

## Iron and the oxidative stress

Iron has also been found to play an important role in oxidative stress. Fenton’s reaction, which causes the conversion of low active H2O2 into potential hydroxyl and peroxide radicals, helps iron ions in ROS production [10,63]. Iron is present in many parts of the body and liver is one of the main sites of storage [64-66], thereby increased iron ions could result in more oxidative stress in liver cells. Usually the concentration of iron in plasma in humans remain stable at 10–30 μM [64] but nearly 40% of CHC patients showed elevated levels of iron and ferritin in serum and 10% of patients showed elevated levels of iron in liver [67]. Researchers found that phlebotomy or dietary iron restriction decreases oxidative stress and lipid peroxidation in CHC patients [68,69].

## Protection

Human body is specially developed to work against oxidative stress [70,71]. The defense system of the body consists of low molecular weight compounds such as glutathione and other antioxidants along with “phase II defense enzymes” that are capable of getting rid of ROS.

### Glutathione

Glutathione is synthesized in all types of eukaryotic cells and especially found in liver. It is considered as one of the most important anti-oxidants. It is also a redox and cell signaling regulator, and works by decreasing H2O2 level and by scavenging reactive oxygen and nitrogen radicals [72]. Researchers found decreased glutathione levels in a large number of CHC patients [26].

Recently, researchers have reported that glutathione, which is usually oxidized in oxidation stress to prevent cellular components from reactive oxygen species, moves towards vacuole by ABC-C transporter Ycf1 rather than staying in the cytoplasm. This movement of the oxidized glutathione to the vacuole protects the cellular metabolic processes of cytoplasm from the oxidative damage. This finding also shows that the conventional methods of determining oxidative stress have to be re-evaluated as the cells could have been under oxidative stress even when the cytoplasm looks healthy [73].

**Figure 2:**
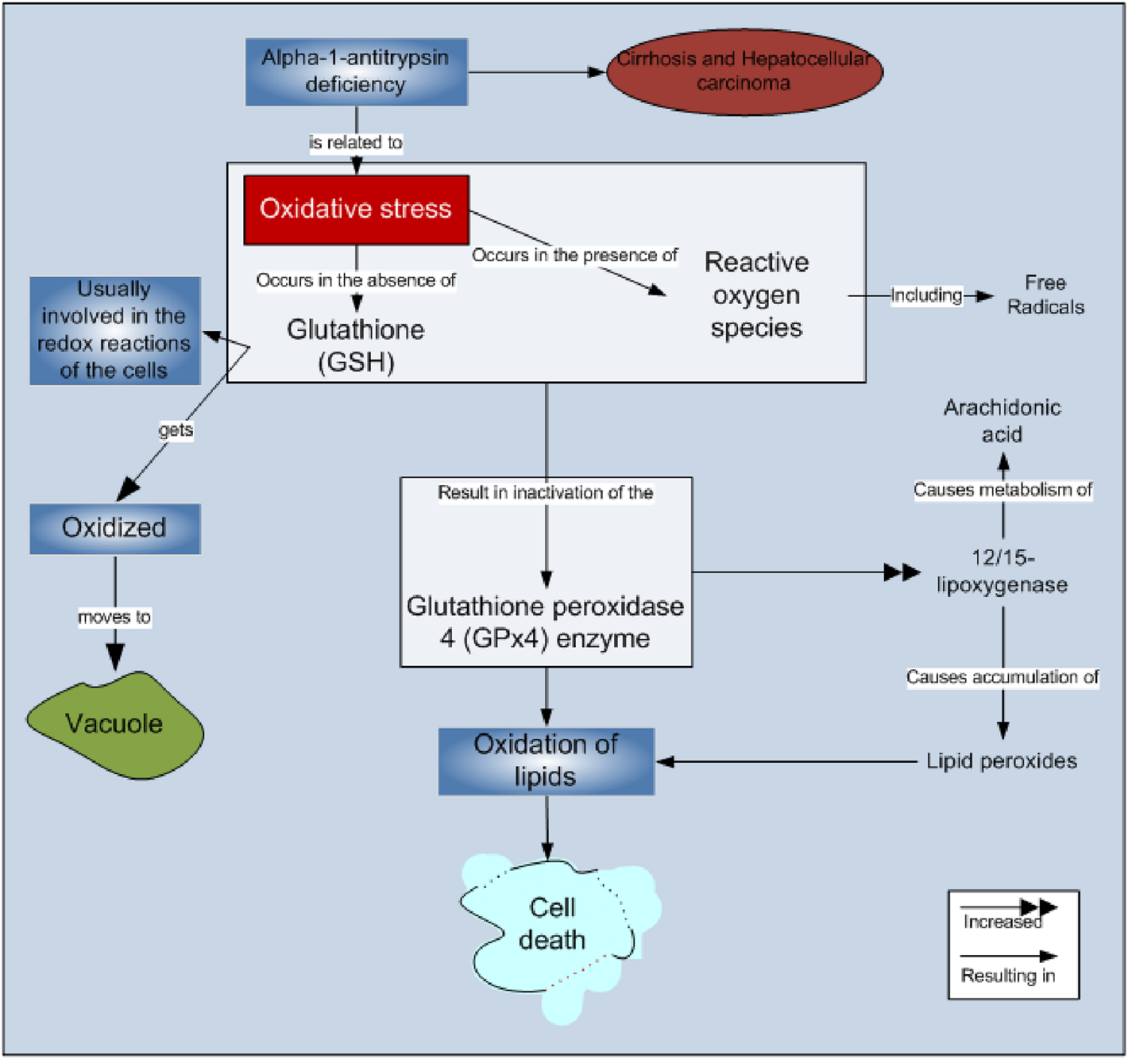
Oxidative stress related processes.

Researchers found that a by-product of glutathione; N-acetylcysteine (NAC), also decreases oxidative stress [74,75]. Some “Oxidative stress” related processes are presented in Figure 2.

### Nrf2

A transcription factor protein, namely Nrf2, controls the cell’s ability to cope with oxidative stress by elevating the expression of key genes for eliminating the damaged proteins. During elevated oxidative stress, Nrf2 increases the production of 20 S proteasome and also affects the Pa28αβ (11 S) proteasome regulator (Pa28) helping in the breakdown of oxidized proteins that could destroy the cells after accumulation [76]. Nrf2 protein has also been found to be related to stem cell division [77]. In another study, this protein has been found to be related to the increased chances of atherosclerosis due to increased plasma cholesterol levels and the cholesterol content in liver [78]. It has been reported that activation of Nrf2, as a result of HCV, is mediated by the mitogen-activated protein (MAP) kinases p38 MAPK and janus kinase [79].

The antioxidant defense Nrf2/ARE pathway is mediated by five viral proteins, i.e., core, E1, E2, NS4B, and NS5A. The core protein is found to be the most potent regulator [80]. However, in another contradictory study, researchers found the suppressed Nrf2/ARE pathway in the HCVcc system [81]. The reasons for this contradiction are not known although the researchers used the similar infection conditions.

### MDA and complement factor H

**Figure 3:**
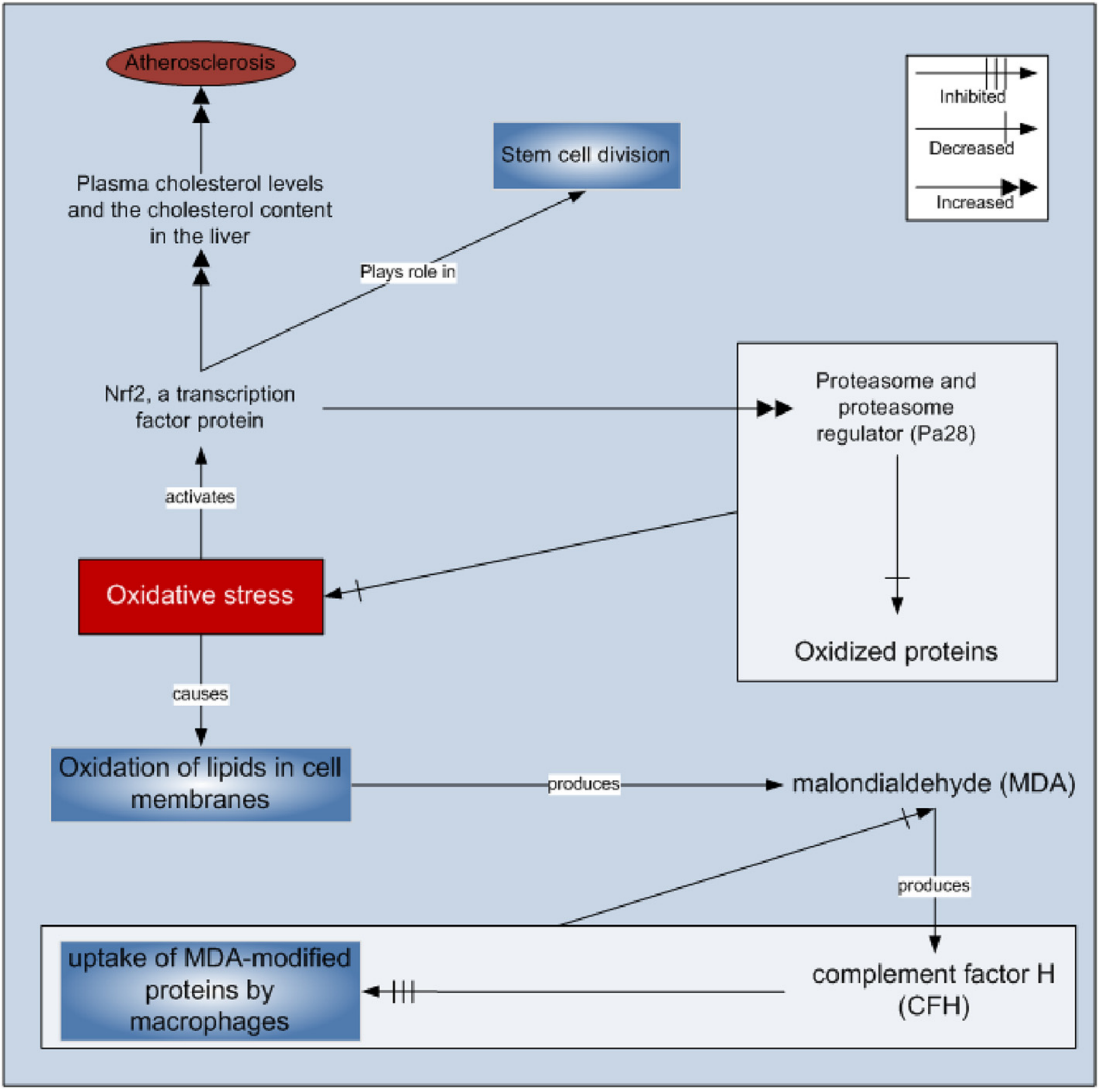
Oxidative stress and self-defense mechanisms.

The lipids in cell membranes produce many of the reactive products upon oxidative stress. One of those compounds is malondialdehyde (MDA) that changes other molecules to produce novel oxidation-specific epitopes that takes the attention and inflammatory reaction of the innate immune system. MDA is also found to attract an immune system protein called complement factor H (CFH) that stops the uptake of MDA-modified proteins by macrophages after binding to MDA. This resulted in neutralization of inflammatory effects of MDA in mice model [82]. Presently known mechanisms of Nrf2 and Complement Factor H are presented in the Figure 3.

### HNF and MRP2

Qadri et al. reported that HCV induces the hepatocyte nuclear factor (HNF)-1 and HNF4, and this process has been attributed to the elevated oxidative stress as well as the direct interaction of NS5A protein and HNF1. Both HNF1 and HNF4 are considered as the important transcriptional factors for the normal development of the liver. This HNF1 activation results in increased MRP (multidrug resistance protein)-2 activity that plays an important role in the detoxification process associated with oxidative stress [83].

### PARP1 and SIRT6

**Figure 4:**
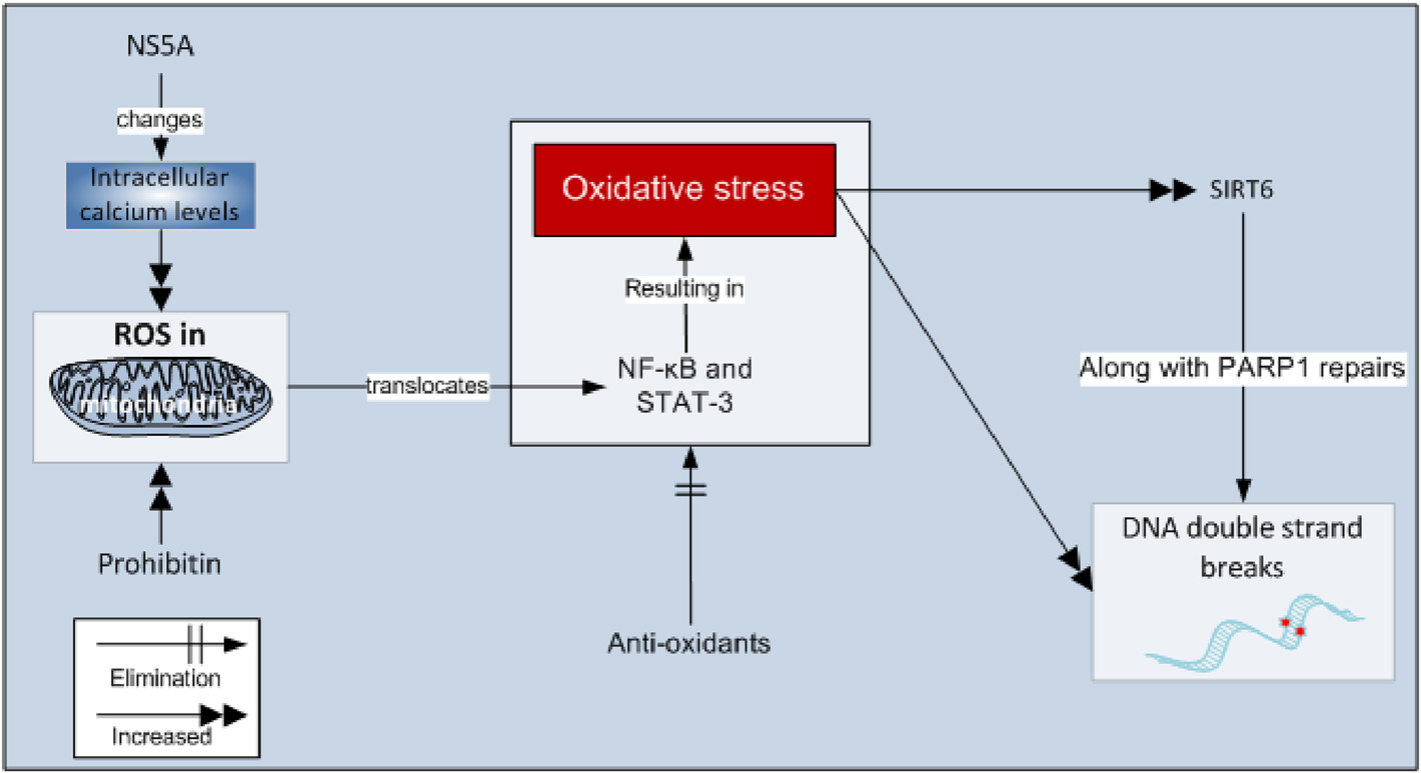
Role of prohibitin and SIRT6 in oxidative stress.

A protein, sirtuin 6 (SIRT6), is produced maximally under oxidative stress. This protein along with poly [adenosine diphosphate (ADP)–ribose] polymerase 1 (PARP1) protein helps in repair of double strand breaks. PARP1 is an enzyme that is among the first compounds responding to DNA damage. SIRT6 is helpful in DNA repair even in the absence of oxidative stress. Researchers found that increased levels of SIRT6 help in more rapid direction of the DNA repair enzymes towards the sites of damage thereby increase the restoration of double strand breaks [84]. The role of prohibitin and SIRT6 are presented in the Figure 4.

## Future directions

There is no doubt that oxidative stress plays an important role in HCV pathogenesis, therefore the combination of several mechanisms described above could be exploited to reach the new solutions of combating the oxidative stress in HCV infection. Antioxidants may be employed in 4 different ways 1) to impair in HCV replication 2) to improve liver enzyme levels, 3) to protect against liver cell damage and 4) to render interferon anti-viral therapy more effective. In fact, triple antioxidants therapies are on rise, which include such as alpha-lipoic acid, silymarin and selenium in suppressing HCV-induced liver disease [85], when used together with Vitamins C and E, and in a healthy diet and exercise regime [86]. Hypolipidemic agent, nordihydroguairetic acid which is also a potential antioxidant has been shown to affect lipid droplet morphology and HCV propagation [87]. MnSOD are shown to be the prime candidates for reversal of HCV-induced fibrosis [88] and activation of NFkB may be another way to boost antioxidant MnSOD response [89]. In a phase II study of HCV patients, the mitochondria-targeted anti-oxidant mitoquinone is shown to decrease liver damage [90].

Researchers showed that peripheral blood leukocyte mtDNA copy number and oxidative stress could help in assessment of mitochondrial damage in HCV-infected patients but it needs further work, whether they can be utilized to evaluate the activity or severity of the HCV-related liver diseases. The clinical significance of damage to mtDNA between the occult and overt HCV-infected patients needs further research. Moreover, the changes in mtDNA damage with time in different HCV-infected patients and the risk of consequent hepatocellular carcinoma needs further studies [13]. Role of oxidative stress on HCV entry and particle assembly and release needs more research in which HCV cell entry is thought to be a multi-step process [91].

Another important thing that needs further research is the role of antioxidant defense systems against HCV as researchers found some contradictory results such as in the case of antioxidant Defense Nrf2/ARE Pathway [26]. Oxidative stress, in mouse model, has also been found to be related to the expression of misfolded human alpha-1-antitrypsin mutant Z protein but the mechanism, which can be more than one, are still not clear. Moreover, the effects of different levels of monomer, non-globular polymer or globular forms on oxidative stress need further research [92].

## Abbreviations

AIF: Apoptosis inducing factor; AP-1: Activator protein-1; ARE: antioxidant response elements; ATM: Ataxia telangiectasia mutated; ATP: Adenosine triphosphate; CHF: Complement factor H; DHCR24: 3β-hydroxysterol Δ24-reductase; ER: Endoplasmic reticulum; GSH: Glutathione; HCC: Hepatocellular carcinoma; HCV: Hepatitis C virus; HNF: Hepatocyte nuclear factor; HO: Heme oxygenase; JNK: c-Jun N-terminal kinase; MDA: Malondialdehyde; MnSOD: manganese-superoxide dismutase; MRP: Multidrug resistance protein; mtDNA: mitochondrial DNA; NO: Nitric oxide; Nox: NADPH oxidase; NS5A: Nonstructural protein 5A; OHCI: occult HCV infection; p38 MAPK: mitogen-activated protein kinase; PARP1: Poly[adenosine diphosphate (ADP)–ribose] polymerase 1; PBMC: peripheral blood mononuclear cells; PERK: PKR-like endoplasmic reticulum kinase; PTK: Protein-tyrosine kinase; RNS: Reactive Nitrogen Species; ROS: Reactive oxygen species; SIRT: Sirtuin; SOD: Superoxide dismutase; TGF-β: Transforming growth factor β; Trx: Thioredoxin.

## Competing interests

The authors declare that they have no competing interests.

## Authors’ contributions

UZP and KF reviewed the literature, and wrote the manuscript. IQ critically reviewed and edited the manuscript. AA, AC, GD and MA helped UZP and KF in literature review. All the authors read and approved the final manuscript.
